# The cellular protein hnRNP A2/B1 enhances HIV-1 transcription by unfolding LTR promoter G-quadruplexes

**DOI:** 10.1038/srep45244

**Published:** 2017-03-24

**Authors:** Matteo Scalabrin, Ilaria Frasson, Emanuela Ruggiero, Rosalba Perrone, Elena Tosoni, Sara Lago, Martina Tassinari, Giorgio Palù, Sara N. Richter

**Affiliations:** 1 Department of Molecular Medicine, University of Padua, Padua, Italy

## Abstract

G-quadruplexes are four-stranded conformations of nucleic acids that act as cellular epigenetic regulators. A dynamic G-quadruplex forming region in the HIV-1 LTR promoter represses HIV-1 transcription when in the folded conformation. This activity is enhanced by nucleolin, which induces and stabilizes the HIV-1 LTR G-quadruplexes. In this work by a combined pull-down/mass spectrometry approach, we consistently found hnRNP A2/B1 as an additional LTR-G-quadruplex interacting protein. Surface plasmon resonance confirmed G-quadruplex specificity over linear sequences and fluorescence resonance energy transfer analysis indicated that hnRNP A2/B1 is able to efficiently unfold the LTR G-quadruplexes. Evaluation of the thermal stability of the LTR G-quadruplexes in different-length oligonucleotides showed that the protein is fit to be most active in the LTR full-length environment. When hnRNP A2/B1 was silenced in cells, LTR activity decreased, indicating that the protein acts as a HIV-1 transcription activator. Our data highlight a tightly regulated control of transcription based on G-quadruplex folding/unfolding, which depends on interacting cellular proteins. These findings provide a deeper understanding of the viral transcription mechanism and may pave the way to the development of drugs effective against the integrated HIV-1, present both in actively and latently infected cells.

G-quadruplexes (G4s) are unique four-stranded nucleic acid structures that may form in guanine-rich sequences. Based on the strand orientation, they can adopt three main topologies: parallel, antiparallel, and hybrid-type structures. G4s have been shown to be involved in key regulatory and pathological roles in eukaryotes, including transcriptional regulation of gene promoters and enhancers, translation, chromatin epigenetic regulation, DNA recombination^1^,^2^,^3^. Formation of G4 *in vivo* has been consolidated by the development of G4 specific antibodies^4^,^5^.

Recently, the presence of G4s in viruses and their involvement in key steps of viral infection has been provided^6^. G4s have been reported in the SARS coronavirus^7^, the human papilloma, hepatitis C, Zika and Ebola virus genomes^8^,^9^,^10^,^11^. Among herpesviruses, RNA G4s have been implicated in the regulation of DNA replication and translation of the Epstein–Barr virus^12^,^13^. We have shown that the herpes simplex virus 1 possesses several repeats of sequences forming stable G4s, which were visualized in infected cells by a G4-specific antibody^14^; stabilization of these tetraplex structures by a G4 ligand inhibited viral DNA replication^15^.

The largest body of evidence of G4-mediated regulation of viruses has been provided for the human immunodeficiency virus-1 (HIV-1), the etiologic agent of the acquired immune deficiency syndrome (AIDS). We and other groups have identified functionally significant G4s in the Nef coding region^16^ and in the unique long terminal repeat (LTR) promoter^17^,^18^,^19^ of HIV-1. When the HIV-1 G4s were stabilized by G4 ligands, antiviral effects, mainly dependent on inhibition of LTR-mediated transcription, were observed^18^,^20^,^21^. Furthermore, the cellular protein nucleolin has been shown to stabilize the HIV-1 LTR G4s and induce potent inhibition of viral transcription^22^.

In general, several proteins that modulate G4s and/or serve as a bridge to recruit additional protein regulators have been reported^23^. Besides the shelterin complex proteins that are involved in telomere homeostasis^24^, G4 interacting proteins either stabilize (e.g. nucleolin, MAZ and nucleophosmin) or unfold (the helicase and heterogeneous nuclear ribonucleoprotein (hnRNP) families)^25^,^26^ the G4 conformation to allow for a tightly controlled modulation of the epigenetic G4 switch.

By means of mass spectrometry, surface plasmon resonance (SPR), fluorescence energy transfer (FRET), Taq polymerase stop and reporter assays, we here identified and characterized G4-selectivity and function of the hnRNP A2/B1, the first protein shown to unfold G4s in the HIV-1 LTR promoter.

## Results

### The human nuclear ribonucleoprotein A2/B1 (hnRNP A2/B1) selectively binds the HIV-1 LTR G-quadruplexes

**Figure 1:**
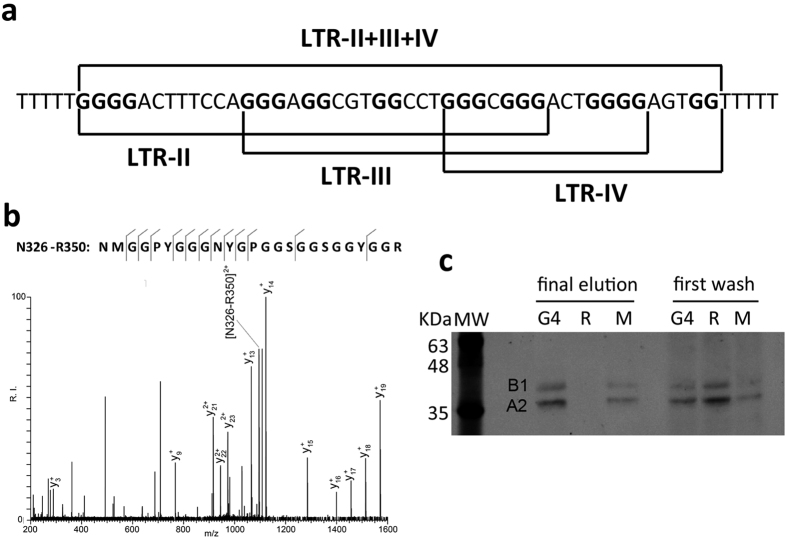
(**a**) LTR-II + III + IV sequence of the G-rich LTR region comprising tracts that fold into three mutually exclusive LTR G4s, i.e. LTR-II, LTR-III and LTR-IV. These three-stacked tetrads G4s are indicated in brackets. G bases in G-tracts or involved in G-quadruplexes are shown in bold. (**b**) MS/MS spectrum of the precursor ion observed at m/z 1095.46 in the sample mixture from digestion of LTR-II + III + IV G4 sample (see Table 1). Only characteristic y ions are indicated^53^. The data match the sequence of peptide N326-R350 of hnRNP A2/B1, which is reported on top with the observed fragments. c) Pull-down assay of nuclear extract proteins with wt, mutant G4 LTR-II + III + IV (M) and random (R) sequences, immobilized on agarose beads. Shown is the western blot analysis with an anti-hnRNP A2/B1 antibody. Proteins complexed to the beads-bound LTRs were washed with augmented stringency by increasing the ionic strength of the wash buffer (0.2 and 1 M). The final elution was obtained in denaturing buffer at 95 °C.

**Figure 2:**
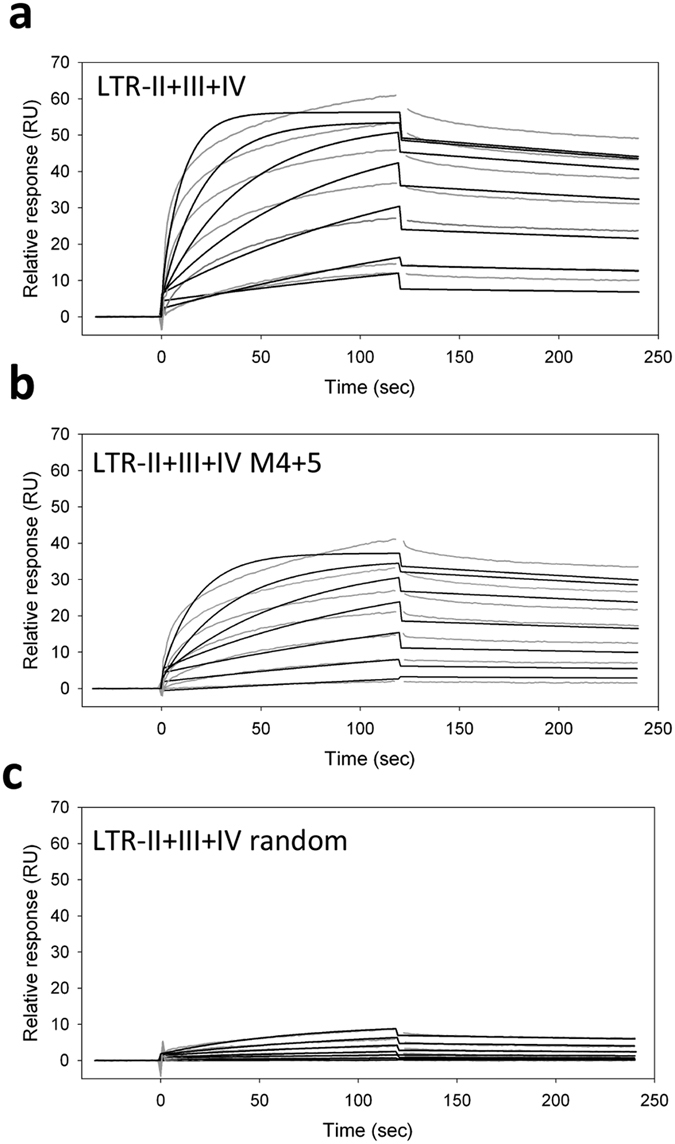
Binding affinity of hnRNP A2 for the LTR-II + III + IV G4 and mutant sequences measured by surface plasmon resonance (SPR) analysis. The human recombinant hnRNP A2 was immobilized on the SPR chip by amine coupling chemistry and the unlabelled oligonucleotides were bound at a flow rate of 25 μl/min, with contact time and dissociation time of 120 s in HEPES-KCl buffer. Sensograms were obtained in the oligonucleotide concentration range of 31.25 nM–2 μM. SPR sensograms of (**a**) LTR-II + III + IV with *k*_*a*_ 4.7 × 10^4^ ± 5.2 × 10^2^ Ms^−1^ and *k*_*d*_ 9.2 × 10^−4^ ± 7.5 × 10^−5^ s^−1^, (**b**) LTR-II + III + IV M4-5 with *k*_*a*_ 2.8 × 10^4^ ± 3.4 × 10^2^ Ms^−1^ and *k*_*d*_ 10.0 × 10^−4^ ± 8.4 × 10^−5^ s^−1^ and (**c**) LTR-II + III + IV Random, the *k*_*a*_ and *k*_*d*_ values of which are not reliably measurable. Sensograms are shown as grey lines and their respective fits as black lines.

We have shown that the HIV-1 LTR promoter can fold into three mutually exclusive G4s and that nucleolin, the most abundant nucleolar protein, can bind, induce and stabilize the LTR G4s^22^. We reasoned that other proteins may exist that regulate the G4/ds equilibrium at the LTR: we thus looked for additional proteins by pull-down assay of 293T nuclear cell extracts against the whole region in the HIV-1 LTR that can fold into G4, i.e. LTR-II + III + IV. This region can alternatively fold into three G4s, i.e. LTR-II, LTR-III and LTR-IV. As control, a two-point mutation LTR-II + III + IV oligonucleotide, which has been previously shown to be unable to fold into G4, i.e. LTR-II + III + IV M4 + 5, was used (Fig. 1a and Supplementary Table S1)^18^. After washing at increasing ionic strength to destabilize weak protein-G4 interactions, the final eluted sample was run on SDS-PAGE and subjected to MS analysis. A series of heterogeneous nuclear ribonucleoproteins (hnRNPs) were found to be present only in the G4-folded oligonucleotide (Table 1); among them, only hnRNP A2/B1 was obtained in all three independent tests, even if it did not obtain the highest score within each experiments (Fig. 1b). The identity and selectivity of hnRNP A2/B1 was confirmed by pull-down assay followed by western blot analysis with an anti-hnRNP A2/B1 antibody. The G4-folded LTR-II + III + IV was used along with the G-rich non-G4 forming LTR-II + III + IV M4 + 5 and a random oligonucleotide of the same length (Supplementary Table S1). The hnRNP A2/B1 protein was confirmed to bind mainly to the G4-folded sequence, even if in these settings it displayed mild recognition also of the G-rich non-G4-folding oligonucleotide (Fig. 1c). To confirm selectivity toward the G4 LTR oligonucleotide, surface plasmon resonance (SPR) analysis was performed. The recombinant purified hnRNP A2, which lacks 12 amino acids in the amino-terminal region and is the main isoform, accounting for ~90% of the protein in most tissues^27^, was immobilized on a SPR chip and binding affinity of the unlabelled oligonucleotides was measured. LTR-II + III + IV displayed the highest K_D_ (19.5 ± 1.5 nM), while the mutant LTR-II + III + IV M4 + 5 was bound with a lower affinity (K_D_ 35.5 ± 3.0 nM). Chi^2^ and U-values were <10 and <15, respectively, indicating optimal data fitting^28^,^29^. In contrast, binding to a random oligonucleotide of the same length as LTR-II + III + IV was so low that in these conditions it was not possible to obtain a meaningful K_D_ value (Fig. 2).

### hnRNP A2 unfolds the LTR G4s

**Figure 3:**
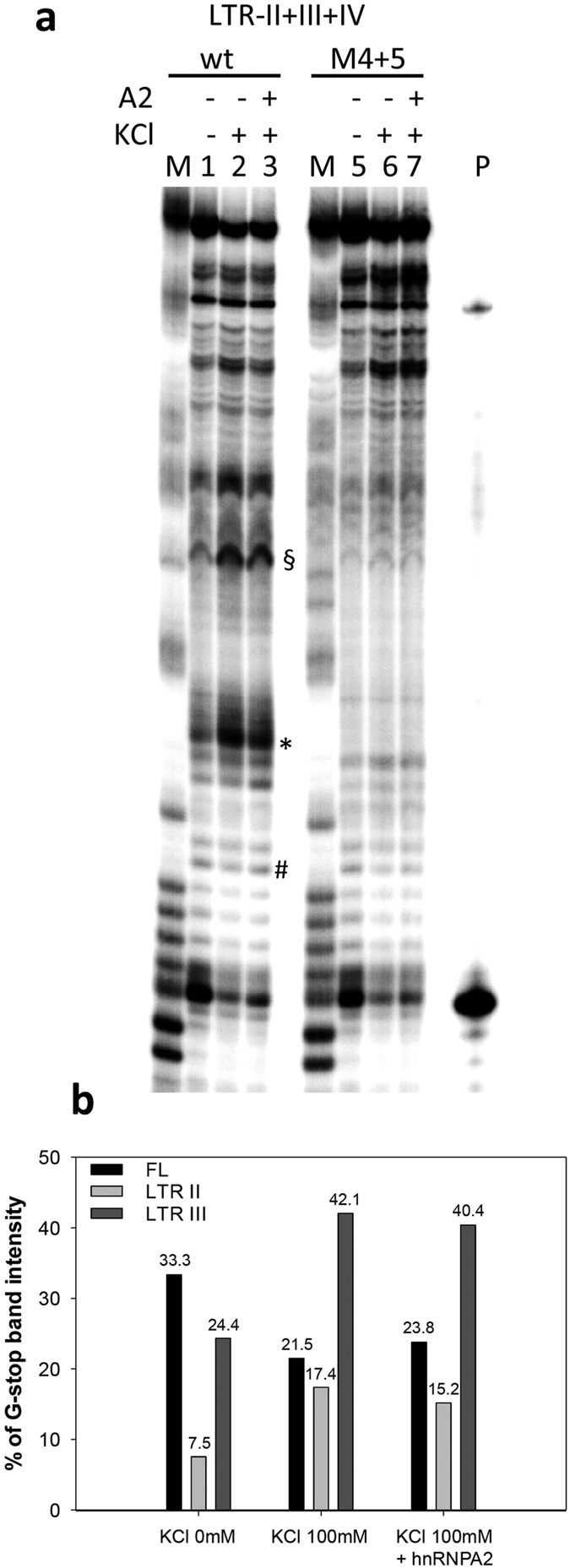
*Taq* polymerase stop assay in the presence of hnRNP A2. (**a**) *Taq* polymerization was performed in the presence/absence of K^+^ and hnRNP A2, as indicated, on the wt and the mutant LTR-II + III + IV M4 + 5 sequences. Amplification of the wt template was performed at 37 °C for 30 min. Stop regions corresponding to LTR-III are indicated by the * symbol, to LTR-II by ^§^ and to LTR-IV by^#^. M is a marker lane obtained with the Maxam & Gilbert sequencing protocol. P indicates the band of the labelled primer. (**b**) Quantification of lanes 1–3 in panel (a). Quantification of stop bands corresponding to formation of LTR-II and LTR-III G4s and of the full-length amplification product (FL) is shown.

We next set out to investigate the effect of hnRNP A2 binding to the LTR G4s. A *Taq* polymerase stop assay was performed on the LTR-II + III + IV template. In the presence of 100 mM K^+^, stop sites corresponding to formation of LTR-III and LTR-II were visible (compare lanes 1 and 2, Fig. 3a). No stop corresponding to LTR-IV was detected, as expected, since LTR-IV has been previously reported to form upon induction by G4 ligands^18^. Upon addition of hnRNP A2, both LTR-III and LTR-II stop sites showed an insignificant decrease (compare lanes 3 and 2, Fig. 3a and b). Similarly, no effect was induced by addition of K^+^ and hnRNP A2 in the control LTR-II + III + IV M4 + 5 template lacking the possibility to form G4 (lanes 5–7, Fig. 3a).

**Figure 4:**
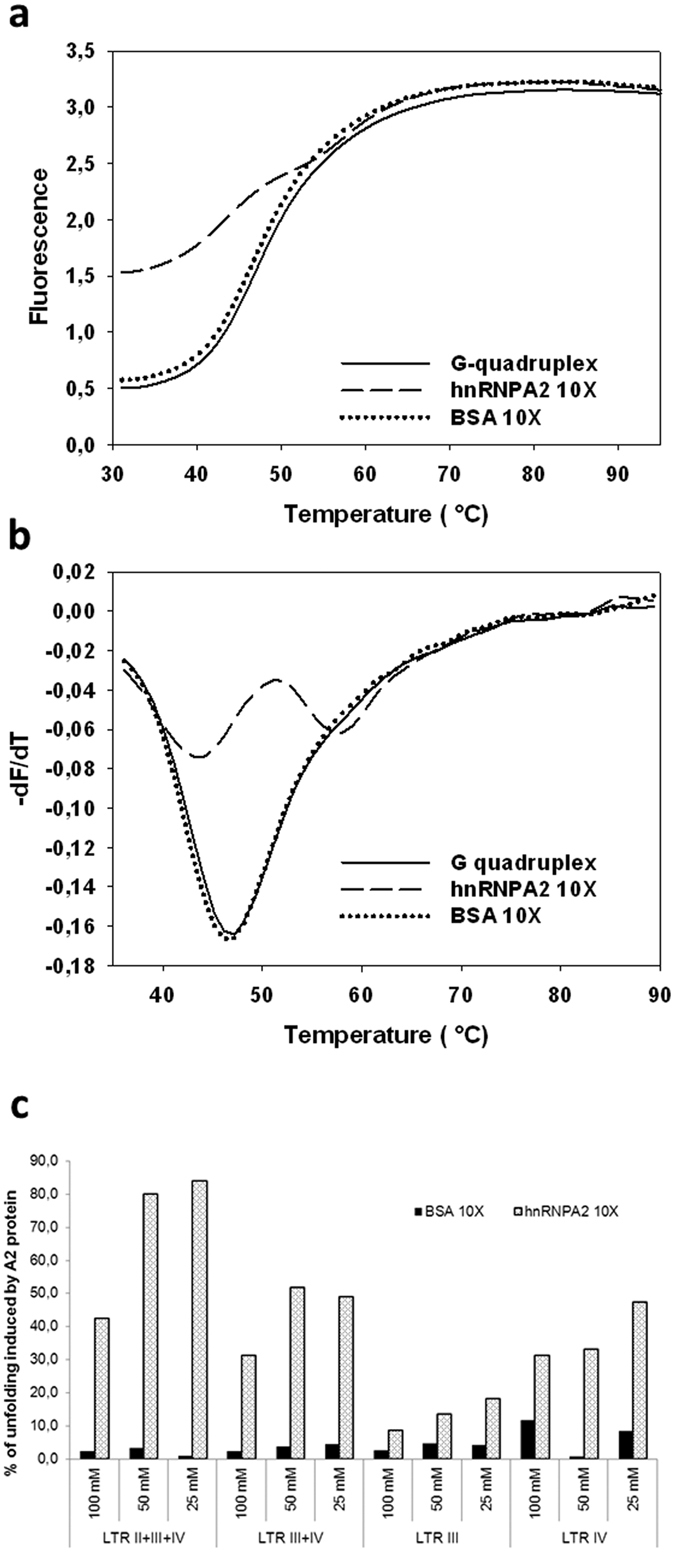
FRET analysis of hnRNP A2 unfolding. (**a**) FRET-melting curves of LTR F-II + III + IV-T, in the absence or presence of hnRNP A2 1:10 or of BSA as control. (**b**) Corresponding first derivative curves, dF/dT versus T. Proteins were incubated with the LTR G4s for 30 min at 37 °C prior to analysis. (**c**) Unfolding % of all tested sequences at 100, 50, 25 mM K^+^, incubated in the presence of hnRNP A2 or BSA at 37 °C for 30 min. Unfolding values are referred to unfolding in the absence of proteins (100%).

As suggested by Xodo^30^, the apparent lack of effect in the presence of an unfolding protein could be due to the protein binding to the template sequence which would stimulate polymerase stop and mask G4 release at the same binding site. We thus switched to a dual-labelled system where oligonucleotide folding could be monitored by changes in fluorescence. Fluorescence resonance energy transfer (FRET) is a spectroscopic technique that provides information about structure and dynamics of nucleic acids folding. It involves a donor fluorophore in an excited state, the excitation energy of which can be transferred to a proximal acceptor chromophore. Given that the major determinant of FRET efficiency is the distance between the acceptor and the donor, fluorescence intensities depict nucleic acids folding states. Consequently, when annealed to its complementary sequence to form a double-stranded structure, the tested oligonucleotide would yield the maximum fluorescence intensity, while the G4 folded conformation would be the least fluorescent. In these conditions the measured fluorescence intensity allows to calculate the energy transfer (E) and the end-to-end distance (R) between the two fluorophores, and therefore the unfolding degree. The G4 folded LTR-II + III + IV sequence was characterized by R of ~38Ǻ, with E ~0.84. When the G4 structure was converted into the duplex conformation by addition of the complementary C-rich strand, the fluorophores were separated by ~150 Ǻ, and FRET was approximately 0. Treatment of LTR-II + III + IV G4 with hnRNP A2 increased fluorescence by 3-folds with respect to the free G4, and E decreased to 0.51 (Fig. 4a and b, Supplementary Table S2). Considering that the complete unfolding of the G4 structure required ΔE = 0.72, the ΔE = 0.30 induced by hnRNP A2 reflected a 42% unfolding (Fig. 4c). The negative control protein BSA showed negligible unfolding activity in these conditions (Fig. 4c and Supplementary Table S2). To test the statistical significance of the effect observed in the presence of the protein, we applied a model comparison approach, which allows to test the statistical role of an independent variable (in our case the hnRNP A2 protein) through the comparison of two models differing only in that variable. The test yielded F (2, 189) = 36.27 and P < 0.05^31^, indicating that the difference observed in the presence and absence of the protein was statistically significant. Analysis of the activity of hnRNP A2 was extended to shorter LTR sequences, i.e. LTR-III + IV, LTR-III and LTR-IV G4s, folded in 100 mM K^+^. The unfolding of LTR-III + IV and LTR-IV was similar but lower than that on the full-length sequence (31%); unfolding of LTR-III was very low (8.5%) (Fig. 4c, Supplementary Table S1 and Supplementary Fig. S2–S4) When the G4-forming sequences were folded in lower K^+^ concentrations, i.e. 50 and 25 mM, a distinct enhancement of unfolding was observed: unfolding up to 83% was obtained in the LTR-II + III + IV oligonucleotide (Fig. 4c, Supplementary Table S1 and Supplementary Fig. S1).

Because G4s are less stable at lower K^+^ concentrations, we proposed that the diverse unfolding efficiency towards the different-length LTR G4s depended on the stability of the LTR G4s. To test this hypothesis, melting temperatures (T_m_) of the dual-labelled oligonucleotides were thus measured by FRET-monitored thermal unfolding (Table 2). The most stable sequence was LTR-III (T_m_ 62.1 ± 0.1 °C), followed by LTR-IV and LTR-III + IV (T_m_ 59.1 ± 0.1 °C and 55.2 ± 0.1 °C, respectively); LTR-II + III + IV was the least stable sequence (T_m_ 49.1 ± 0.1), confirming that the observed unfolding scale depended on the oligonucleotide stability in the FRET system. To note that in the unlabelled oligonucleotides measured by circular dichroism (CD), LTR-IV was the least stable sequence (Table 2), bias which likely derives by the absence of fluorophores at the oligonucleotides’ 5′- and 3′-end, which affect T_m_ values, as previously noted^32^.

**Figure 5:**
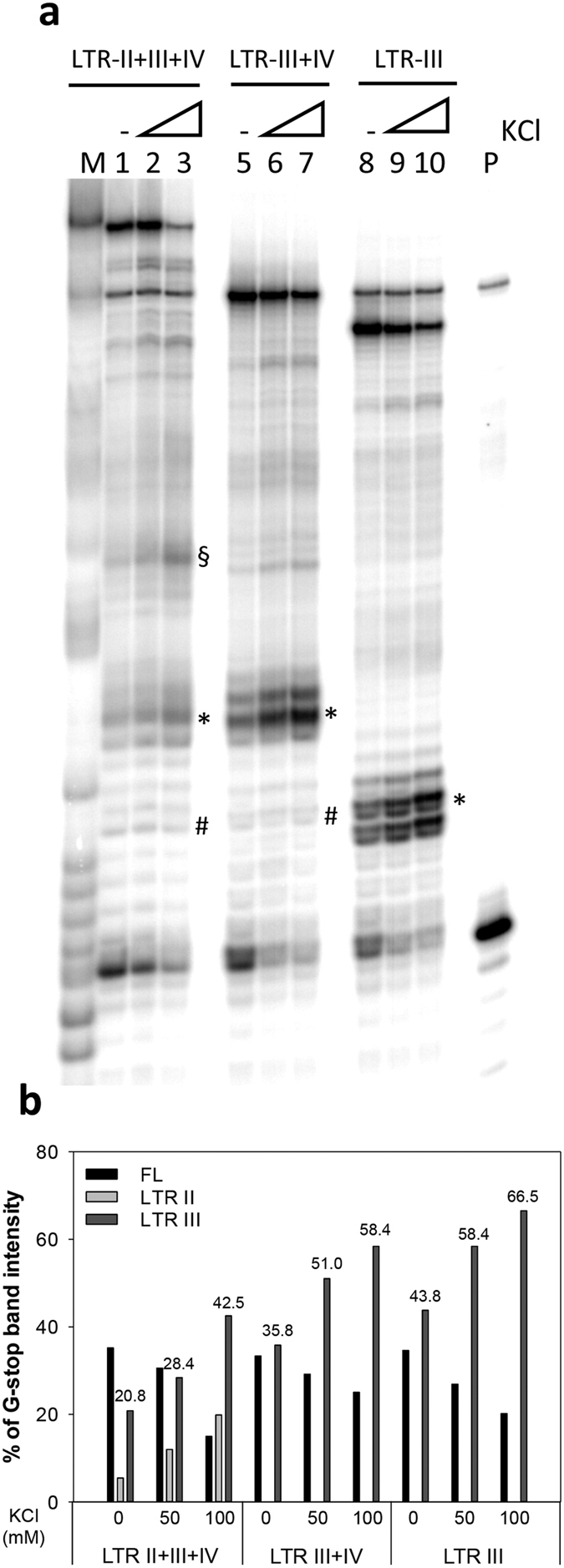
*Taq* polymerase stop assay at increasing K^+^ concentrations. (**a**) Oligonucleotides were folded in the absence or presence of K^+^ (50–100 mM). Oligonucleotides were used as templates in a Taq polymerase reaction at 37 °C. Stop regions corresponding to LTR-III are indicated by the *symbol, to LTR-II by § and to LTR-IV by^#^. P indicates the band of the labelled primer. M is a marker lane obtained with the Maxam & Gilbert sequencing protocol. (**b**) Quantification of lanes 1–10 of panel (a). Quantification of stop bands corresponding to formation of LTR-II and LTR-III G4s and of the full-length amplification product (FL) is shown.

In addition, the longest sequences, i.e. LTR-II + III + IV and LTR-III + IV, where multiple G4s may form, display T_m_ values that represent the average stability, which depends on the effective formation of the possible G4s. We thus performed a *Taq* polymerase stop assay to assess the G4 mutual formation and stability at increasing K^+^ concentration in the full-length LTR. LTR-III was used as a control sequence where only one G4 could form. We observed that in the full-length LTR-II + III + IV sequence, both LTR-II and LTR-III G4s formed upon addition of K^+^ (lanes 2–3, Fig. 5a); of these, LTR-III G4 induced a slightly more intense stop than LTR-II, suggesting a preferred formation of LTR-III in the full-length sequence (Fig. 5b). LTR-III was also the only G4 forming in the LTR-III + IV template (lanes 5–7, Fig. 5a). Interestingly, the LTR-III that formed in the full-length sequence was less intense than LTR-III forming in the shortest oligonucleotides (compare lanes 2–3, with 6–7 and 9–10, Fig. 5a and b). These data indicate that i) LTR-III is the most prominent G4 among the mutually exclusive LTR G4s and ii) when LTR-III forms in the full-length LTR promoter, its stability maintains a level that is fully susceptible to hnRNP A2 processing.

### Depletion of hnRNP A2/B1 decreases viral transcription

**Figure 6:**
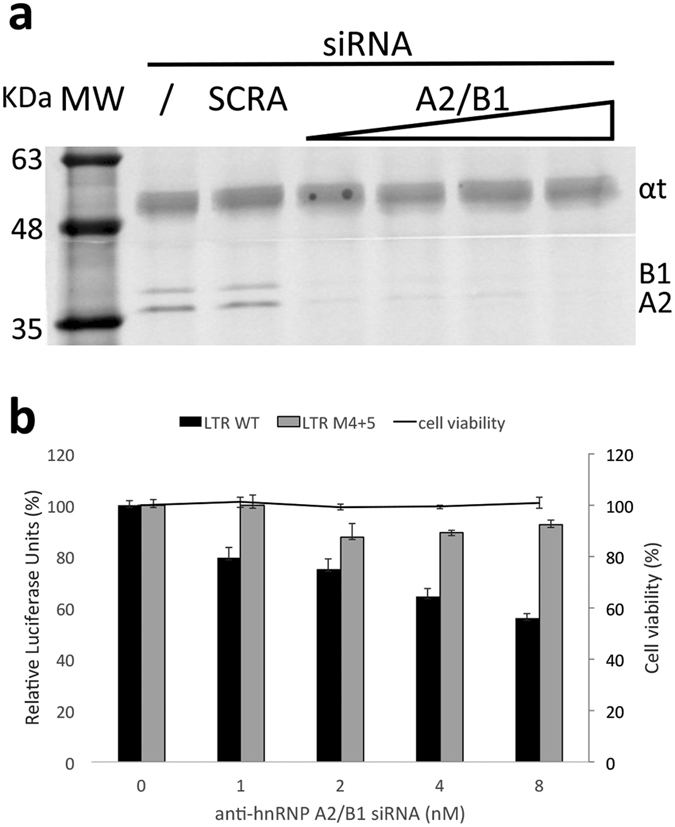
Effect of hnRNP A2/B1 on the HIV-1 LTR promoter in cells. (**a**) hnRNP A2/B1 depletion in 293T cells by siRNAs analysed by western blot with anti hnRNP A2/B1 antibody. Scra indicates scrambled siRNAs. Detection of α-tubulin (αt) was used as control. The symbol“/”indicates untreated cells. (**b**) Analysis of the luciferase activity of the wt and M4 + 5 LTR promoters in 293T cells treated with hnRNP A2/B1 siRNAs, normalized to protein content.

We have shown that LTR G4 folding inhibits viral transcription, whereas when point mutations that disrupt G4s are introduced in the LTR promoter, an increase in promoter activity is evidenced^18^. Silencing of the LTR G4 folding/stabilizing protein nucleolin significantly increased promoter activity, indicating the inhibitory effect of nucleolin on LTR-driven transcription^22^. To assess the effect of hnRNP A2/B1 binding/unfolding of the full-length LTR G4s, a set of anti-hnRNP A2/B1 siRNAs was used, which effectively silenced hnRNP A2/B1 expression in 293T cells (Fig. 6a). In these conditions, LTR-driven transcription decreased by 45% at the highest siRNA concentration in the wt LTR sequence (Fig. 6b). In contrast, the LTR sequence with two point mutations that disrupt G4 folding was only marginally affected by hnRNP A2/B1 depletion (Fig. 6b), indicating that the effect was G4-specific. In these conditions, hnRNP A2/B1-silenced 293T cells showed no reduction in cell viability at 48 h with respect to the controls, thus confirming the consistency of the inhibitory effects on LTR promoter activity. Silencing of hnRNP A2/B1 was also performed in the TZM-bl reporter cell line, which contains stably integrated copies of the luciferase gene under control of the LTR wt promoter and thus closely resembles the integrated provirus. The LTR-driven reporter transcription decreased up to 33% in hnRNP A2/B1-silenced cells (Supplementary Fig. S5). These data further confirm the unfolding activity of hnRNP A2/B1 on the LTR G4s.

## Discussion

We have shown that hnRNP A2/B1 selectively binds the HIV-1 LTR G4s and unfolds them. While hnRNPs are RNA/protein complexes that bind newly synthesized RNA (pre-mRNA) in the cell nucleus during gene transcription and post-transcriptional modifications^33^, two of the most abundant of them, hnRNP A1 and hnRNP A2/B1, have been reported to bind also DNA in the quadruplex conformation. Initially hnRNP A1 was found to unfold the telomeric G4s and promote telomerase activity^34^,^35^,^36^, to unfold the G4 in the promoter of the KRAS oncogene and regulate transcription^30^ and to destabilize the tetraplex forms of the fragile X expanded sequence d(CGG)n^37^. HnRNP A2 has been shown to unfold the fragile X repeats^38^ and the shorter hnRNP A2 splice variant to promote telomere extension in mammalian cells^39^.

Our finding that hnRNP A2 binds and unfolds the G4s in the promoter of HIV-1 not only confirms the DNA G4 unfolding activity of this protein, but also indicates for the first time that this type of activity is exploited by a virus.

In HIV-1, hnRNP A2/B1 has been reported to play with Rev an important accessory role in promoting nuclear viral RNA retention and nucleocytoplasmic viral RNA transport^40^. In this context, the lower virus production observed upon siRNA-mediated depletion of hnRNP A2/B1 in infected cells was ascribed to accumulation of viral genomic RNA in cytoplasmic compartments or in the nucleus^41^. Our results showing decreased transcriptional activity upon depletion of hnRNP A2/B1 indicate a possible additional mechanism of inhibition of virus production mediated by increased G4 folding in the HIV-1 LTR promoter in the absence of hnRNP A2/B1. Surprisingly, overexpression of this protein and other hnRNP members induced similar effects, i.e. inhibition of transcription and reduction of virus production^42^. We suggest that upon stimuli that boost viral transcription (i.e. integral release of LTR G4s upon hnRNP A2/B1 massive overexpression) the virus (or the cell) activates counteracting mechanisms to avoid excessive exploitation of the cell by the virus that would lead to fast cell death, in the end impairing virus production. Therefore, the reported transcription inhibition might be mediated by other factors triggered by hnRNP A2/B1 overexpression.

The activity of hnRNP A2/B1 is fit to unfold the G4s that actually form in the HIV-1 LTR region. In fact, G4s folded in short oligonucleotides, such as LTR-III and LTR-IV, which displayed higher thermal stability, were only partially unfolded by the protein. In contrast, the same G4 structures folding in a longer context (LTR-II + III + IV) were less stable and were effectively processed by hnRNP A2/B1. Obviously, the longer oligonucleotide better mimes the actual G4 condition in the full-length LTR, as also proved by transcription inhibition obtained by hnRNP A2/B1 depletion in cells that contained the entire HIV-1 LTR.

The LTR G4 system displays regulating features similar to those described for the c-myc^43^,^44^, HRAS^26^ and KRAS oncogene-promoters^30^,^45^,^46^,^47^. As in these eukaryotic G4-modulated promoters, the HIV-1 LTR promoter is processed by G4 stabilizing (nucleolin)^22^ and destabilizing proteins (hnRNP A2/B1). Because the LTR promoter has been suggested to be the region where viral latency is regulated^48^,^49^, the G4 switch may play a role not only in activation of effective viral transcription, but also in its shift to latency. Currently, only the actively transcribed virus is targeted by antiviral drugs, while eradication of the HIV-1 infection has been made impossible by the existence of reservoirs of the latent virus^50^. Therefore, our findings not only advance our understanding on the mechanism of viral transcription but may also constitute a progress from a therapeutic point of view.

## Methods

### Oligonucleotides and cells

All desalted oligonucleotides were purchased from Sigma-Aldrich, Milan, Italy (Supplementary Table S1). TZM-bl (obtained through the NIH AIDS Reagent Program, Division of AIDS, NIAID, NIH, from Dr. J.C. Kappes, Dr. X. Wu, and Tranzyme Inc.) is a HeLa cell line stably expressing large amounts of CD4 and CCR5 and containing integrated copies of the luciferase and β-galactosidase genes under control of the HIV-1 promoter. Human embryonic kidney (HEK) 293T (ATCC # CRL-3216) and TZM-bl cells were grown in DMEM (Gibco, Thermo Fisher Scientific, Waltham, MA, USA) supplemented with 10% heat-inactivated fetal bovine serum (FBS, Gibco, Thermo Fisher Scientific, Waltham, MA, USA). Cells were grown in a humidified incubator maintained at 37 °C with 5% CO_2_.

### Protein nuclear extraction and pull-down assay

Protein nuclear extracts of HEK 293T cells were obtained by using NXTRACT kit (Sigma-Aldrich, Milan, Italy). HEK 293T protein nuclear extracts (150 μg) were incubated with biotinylated LTR-II + III + IV G4, mutated M4 + 5 and random R oligonucleotides folded (600 nM) in 250 μl of reaction containing Tris-HCl 20 mM, pH 8, KCl 30 mM, MgCl_2_ 1.5 mM, protease inhibitor cocktail (Sigma-Aldrich, Milan, Italy) 1%, NaF 5 mM, Na_3_VO_4_ (Sigma-Aldrich, Milan, Italy) 1 mM, poly [dI-dC] (Sigma-Aldrich, Milan, Italy) 1.25 ng/μl for 2 h at 37 °C. The binding reaction was followed by incubation (2 h at 37 °C) with 30 μl of streptavidin-agarose beads (Sigma-Aldrich, Milan, Italy). After PBS and NaCl washes (0.2 and 1 M), beads were collected by brief centrifugation, resuspended in 50 μl of Laemmli buffer, and finally incubated at 95 °C for 5 min. Supernatants were separated in 12% SDS-PAGE and, after coomassie staining, the gel lanes of LTR G4 and LTR M4 + 5 were directly in-gel digested and analysed for protein identification by mass spectrometry (MS) (see MS protein identification in ref. 22). Briefly, gel lanes cut in ~0.5 cm pieces were first washed with 50% CH_3_OH and 2.5% acetic acid, dehydrated with CH_3_CN, then reduced with 30 μl of DTT (10 mM in 100 mM NH_4_HCO_3_) for 30 min at room temperature; the excess of DTT was next neutralized by alkylation with 30 μl of iodoacetamide (50 mM in 100 mM NH_4_HCO_3_) for 30 min at room temperature. Bands were washed with 100 mM NH_4_HCO_3_, dehydrated with CH_3_CN twice, and then digested overnight with 1 μg of MS-grade trypsin (ThermoFisher Scientific, Waltham, MA, USA) in 50 μl of 50 mM NH_4_HCO_3_. Peptides were extracted twice with 5% formic acid and twice with 50% CH_3_CN/5% formic acid. The peptide mixture was further desalted in a silica nanocolumn (Polymicro Technologies, Phoenix, AZ, USA) packed in house with pinnacle C18 pack material (Thermo Fisher Scientific, Waltham, MA, USA). The desalted mixture was finally analyzed by direct infusion electrospray ionization (ESI) on a Thermo Fisher Scientific (Waltham, MA, USA) LTQ-Orbitrap Velos mass spectrometer utilizing quartz emitters produced in house. A stainless steel wire was inserted through the back-end of the emitter to supply an ionizing voltage that ranged between 0.8 and 1.2 kV. Putative peptides present in wt LTR-II + III + IV (but not in M4 + 5) samples were submitted to tandem mass spectrometric (MS/MS) analysis. The masses of the 50 most intense fragment ions were employed to perform a Mascot Database Search^51^ to identify their parent protein. Significant Mascot hits were accepted as positive matches and their ion score reported (see MS protein identification in ref. 22 for more details). Supernatants were further analysed by Western blot with an anti-hnRNP A2/B1 antibody (mouse monoclonal DP3B3; Santa Cruz Biotechnology, Dallas, TX, USA).

### Surface plasmon resonance (SPR) analysis

SPR was performed on the Biacore T100 platform (GE Healthcare, Life Science, Milan, Italy). About 1000 RU of human recombinant hnRNP A2 (Origene Technologies, Rockville, USA) were immobilized on Serie S sensor chip CM5 by amine coupling chemistry. Immobilization was performed in HEPES-NaCl running buffer (HEPES pH 7.4 10 mM, NaCl 150 mM, EDTA 3 mM). Flow cell 1 was blank immobilized to permit reference subtraction. Binding analysis of LTR-II + III + IV, LTR-II + III + IV M4 + 5 and LTR-II + III + IV Random sequences was performed at a flow rate of 25 μl/min, with contact time and dissociation time of 120 s in HEPES-KCl buffer (HEPES pH 7.4 10 mM, KCl 150 mM, EDTA 3 mM). Oligonucleotides folding into G4 structures was performed in HEPES-KCl buffer after heat denaturation at 95 °C for 5 min and gradual cooling at room temperature. Sensograms were obtained in the concentration range of 31.25 nM–2 μM. After each oligonucleotide injection the chip surface was regenerated with NaCl 2.5 M solution. All sensograms were corrected by double reference subtraction of blank flow cell response and buffer injection response. Data were fitted to a global 1:1 binding model using BIAevaluation software (GE Healthcare).

### FRET-melting assay

Oligonucleotides were diluted to 0.1 μM in lithium cacodylate buffer (10 mM, pH 7.4) and KCl (indicated in figure legends) heat denatured for 5 min at 95 °C, and folded in G-quadruplex structure at room temperature for 16 h. Samples were incubated alone, with hnRNP A2 or bovine serum albumin (BSA, negative control) and subsequently fluorescence intensity was monitored from 30 °C to 95 °C (1 °C/min) in a LightCycler II (Roche, Milan, Italy) by observing 6-carboxyfluorescein (6*FAM*) emission. FRET efficiency (E) was calculated as the total fluorescence intensity of the donor in the absence (I_D_) and presence of the acceptor (I_DA_), by using E = 1 − (I_DA_/I_D_). I_D_ and I_DA_ values were obtained on a LS 55 Fluorescence spectrometer (Perkin Elmer, Waltham, MA, USA) setting the excitation wavelength at 480 nm and recording the emission from 500 to 650 nm. The dual-labelled oligonucleotide was converted into the corresponding duplex, in which the fluorophores are at a distance (R) for which FRET is ~0. R was calculated from FRET efficiency values as 
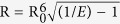
, where R_0_ (Förster distance) is the distance at which energy transfer is 50% of the maximum value. Between FAM and TAMRA fluorophores, R_0_ is assumed to be 50Ǻ^26^. F-test (F) and the probability (P) values were calculated using R statistical environment (v. 3.3.2)^31^. A conventional alpha = 0.05 was considered to evaluate the test significance.

### Taq Polymerase Stop Assay

*Taq* polymerase stop assay was carried out as previously described^18^,^22^. Briefly, the 5′-end labelled primer was annealed to its template (Table S1) in lithium cacodylate buffer in the presence or absence of KCl (50–100 mM) and by heating at 95 °C for 5 min and gradually cooling to room temperature. Where specified, samples were incubated with 29 ng of human recombinant hnRNP A2 (Origene Technologies, Rockville, USA) at 37 °C for 15 min. Primer extension was conducted with 2 U of AmpliTaq Gold DNA polymerase (Applied Biosystem, Carlsbad, California, USA) at 37 °C for 30 min. Reactions were stopped by ethanol precipitation, primer extension products were separated on a 15% denaturing gel, and finally visualized by phosphorimaging (Typhoon FLA 9000).

### Circular Dichroism Spectroscopy

DNA oligonucleotides were diluted to a final concentration (4 μM) in lithium cacodylate buffer (10 mM, pH 7.4) and KCl 100 mM. Samples were annealed by heating at 95 °C for 5 min and gradually cooled to room temperature overnight. CD spectra were recorded on a Chirascan-Plus (Applied Photophysisics, Leatherhead, UK) equipped with a Peltier temperature controller using a quartz cell of 5 mm optical path length, over a wavelength range of 230–320 nm. For the determination of *T*_m_, spectra were recorded over a temperature range of 20–90 °C, with temperature increase of 5 °C. The reported spectra are baseline-corrected for signal contributions due to the buffer. Observed ellipticities were converted to mean residue ellipticity (θ) = deg × cm^2^ × dmol^−1^ (mol ellip). *T*_m_ values were calculated according to the van’t Hoff equation, applied for a two-state transition from a folded to unfolded state, assuming that the heat capacity of the folded and unfolded states are equal^52^.

### Immunoblot analysis

Immunoblot analysis was performed on cell protein extracts obtained in RIPA Buffer (20 mM Tris-HCl pH 7.5; 150 mM NaCl, 1 mM EDTA, 1% NP-40, 1% sodium deoxycholate, 1 mM Na_3_VO_4_, 1x protease inhibitors). Protein concentrations were quantified using the Pierce^®^ BCA Protein Assay Kit (Thermo Scientific, Rockford, IL, USA). Each sample was electrophoresed on 10% SDS-PAGE and transferred to a nitrocellulose blotting membrane (Amersham TM Protan TM, GE Healtcare Life science, Milan, Italy) by using trans-blot SD semi-dry transfer cell (Bio-Rad Laboratories, Milan, Italy). The membranes were blocked with 2.5% skim milk in PBST (0.05% Tween 20 in PBS). Membranes were incubated with the respective primary antibody directed against hnRNP A2/B1 (mouse monoclonal; Santa Cruz Biotechnology, Dallas, TX, USA), alpha-tubulin (mouse monoclonal; Sigma-Aldrich, Milan, Italy). After three washes in PBST, membranes were incubated with ECL Plex Goat-α-Mouse IgG-Cy5 (GE Healthcare Life sciences, Milan, Italy). Images were captured on the Typhoon FLA 9000.

### siRNA and luciferase reporter assay

Gene-specific pooled siRNA trilencer targeting human hnRNP A2/B1 and a scrambled negative control duplex were purchased from Origene (human hnRNP A2/B1 Trilencer-27 Human siRNA, OriGene Technologies, Rockville, MD, USA). 293T cells or TZM-bl reporter cells were transfected with increasing concentrations of siRNA (1, 2, 4 and 8 nM for 293T and 1, 5, 10 nM for TZM-bl cells) and scrambled siRNA by using Lipofectamine RNAiMAX (Invitrogen, Thermo Fisher Scientific, Waltham, MA, USA) following the manufacturer’s instructions. Only for experiments in 293T cells, pLTR luciferase plasmids (pGL4.10-LTRwt or pGL4.10-LTR-M4 + 5) were transfected 24 h later by using Lipofectamine 3000 (Invitrogen, Thermo Fisher Scientific, Waltham, MA, USA). Luciferase activity was measured using the britelite plus Reporter Gene Assay System (PerkinElmer Inc., Milan, Italy) at a Victor X2 multilabel plate reader (PerkinElmer Inc., Milan, Italy), according to the manufacturer’s instructions. Cells were lysed in 1% Triton-X100-PBS and protein concentration was determined by BCA assay (Thermo Scientific Pierce, Monza, Italy). Luciferase signals were subsequently normalized to total protein content, according to the manufacturer’s protocol (http://ita.promega.com/~/pdf/resources/pubhub/cellnotes/normalizing-genetic-reporter-assays/). Each assay was performed in duplicate and each set of experiments was repeated at least three times.

### Cell Viability Assay

The MTT (3-(4,5-dimethylthiazol 2-yl)-2,5-diphenyltetrazolium bromide, Sigma Aldrich, Milan Italy) assay was performed to assess cell viability on silenced cells. 293T cells (6 × 10^3^) were plated in 96-well plates and incubated for 24 h. Following siRNA transfection (from 1 nM to 8 nM), cells were incubated for an additional 48 h. Control cells (transfected in absence of siRNA) were treated in the exact same conditions. Cell survival was evaluated by MTT assay: 10 μL of freshly dissolved solution of MTT (5 mg/mL in PBS) were added to each well, and after 4 h of incubation, MTT crystals were solubilized in solubilization solution (10% sodium dodecyl sulphate (SDS) and 0.01 M HCl). After overnight incubation at 37 °C, absorbance was read at 540 nm. Data were expressed as mean values of at least two individual experiments conducted in triplicate. The percentage of cell survival was calculated as follows: cell survival = (A_well_ − A_blank_)/(A_control_ − A_blank_) × 100, where blank denotes the medium without cells.

## Additional Information

**How to cite this article**: Scalabrin, M. *et al*. The cellular protein hnRNP A2/B1 enhances HIV-1 transcription by unfolding LTR promoter G-quadruplexes. *Sci. Rep.* **7**, 45244; doi: 10.1038/srep45244 (2017).

**Publisher's note:** Springer Nature remains neutral with regard to jurisdictional claims in published maps and institutional affiliations.

## Supplementary Material

Supplementary Information

## Figures and Tables

**Table 1:** MS analysis of protein content of three independent experiments and collected from SDS gels.

Exp#	Experimental monoisotopic mass	Expected monoisotopic mass	Protein match	Peptide match	Score^*^
1	1921.102	1921.079	Putative zinc finger protein 812	V145-R162	18
1,2, 3	2188.904 2219.053	2188.898 2219.063	Heterogeneous nuclear ribonucleoprotein A2/B1	N326-R350 D130-R147	27 18
1	2429.146	2429.157	Heterogeneous nuclear ribonucleoprotein D0	I198-K218	31
2	1681.900	1681.907	Heterogeneous nuclear ribonucleoprotein C1/C2	M74-K89	39
2	863.487	863.487	Mediator of RNA polymerase II transcription subunit 11	V97-K103	22
2	1036.698	1036.676	Histone H1.2	A192-K201	20
2	1036.698	1036.687	Transcription factor AP-4	R294-R302	18
3	952.346	952.349	Cysteine/serine-rich nuclear protein 2	D249-R257	16
3	3450.685	3450.699	Non-POU domain-containing octamer-binding protein	G399-R434	28

*The score (assigned on probabilistic bases by mascot software, “ http://www.matrixscience.com/help/interpretation_help.html#THRESHOLDS”)^51^ is the probability that the observed match is not a random event. The score is reported as −10x log10(P) where P is the absolute probability. The mass of the putative peptides and of the 50 most intense fragment ions were used for the data base search. Significant Mascot hits were accepted as positive matches and their ion score reported. The highest score obtained in the three experiments is reported.

**Table 2:** Melting temperatures (T_m_) of LTR G4s measured on dual-labelled oligonucleotides by FRET and on unlabelled oligonucleotides by CD.

LTR	K^+^ (mM)	T_m_ (°C)	
		FRET	CD
**II** + **III** + **IV**	*100*	49.1 ± 0.1	61.1 ± 0.2
	*50*	45.1 ± 0.1	nd
	*25*	43.2 ± 0.1	nd
**III** + **IV**	*100*	55.2 ± 0.1	64.6 ± 0.1
	*50*	49.1 ± 0.1	nd
	*25*	46.2 ± 0.1	nd
**III**	*100*	62.1 ± 0.1	68.7 ± 0.5
	*50*	56.0 ± 0.1	nd
	*25*	52.1 ± 0.1	nd
**IV**	*100*	59.1 ± 0.1	50.6 ± 0.8
	*50*	57.1 ± 0.1	nd
	*25*	51.1 ± 0.1	nd

T_m_ values are reported with standard deviation. Nd stands for “not-determined”.
