# Supplementary material for: The cellular protein hnRNP A2/B1 enhances HIV-1 transcription by unfolding LTR promoter G-quadruplexes
Source: Sci Rep. 2017 Mar 24;7:45244. doi: 10.1038/srep45244 (PMC5364415; doi:10.1038/srep45244)

The cellular protein hnRNP A2/B1 enhances HIV-1 transcription by unfolding LTR promoter G-quadruplexes

Matteo Scalabrin+, Ilaria Frasson+, Emanuela Ruggiero+, Rosalba Perrone, Elena Tosoni, Sara Lago, Martina Tassinari, Giorgio Palù, Sara N. Richter*

Department of Molecular Medicine, University of Padua, Padua, Italy

*sara.richter@unipd.it

+These authors contributed equally to this work

Supplementary Material Legend

Table S1. Oligonucleotides used in this study.

Table S2. Energy Transfer, Energy Transfer Difference and Radius (Å) of LTRs in the absence or presence of hnRNP A2 or BSA, at different K^+ concentrations.

Fig. S1. FRET-melting curves of LTR-II+III+IV, in the absence or presence of hnRNP A2 or BSA.

Fig. S2. FRET-melting curves of LTR-III+IV, in the absence or presence of hnRNP A2 or BSA.

Fig. S3. FRET-melting curves of LTR-III, in the absence or presence of hnRNP A2 or BSA.

Fig. S4. FRET-melting curves of LTR-IV, in the absence or presence of hnRNP A2 or BSA.

Fig. S5. Activity of hnRNP A2/B1 on the HIV-1 LTR promoter in TZM-bl cells.

Table S1. Oligonucleotides used in this study.

Assay	Name	Sequence 5'-3'
Pull Down	LTR-II+III+IV	d(TTTTTGGGGACTTTCCAGGGAGGCGTGGCCTGGGCGGGACTGGGGAGTGGTTTTT-*BtnTg*)
	LTR-II+III+IV M4+5	d(TTTTTGGGGACTTTCCAGGGAGGCGTGGCCTGTGCGTGACTGGGGAGTGGTTTTT-*BtnTg*)
	Random	d(AAAAACTACTGCACGCTCGCTACGACGACACTGTCGCGCATACAAGCTGCAAAAA-BtnTg)
SPR	LTR-II+III+IV	TTTTTGGGGACTTTCCAGGGAGGCGTGGCCTGGGCGGGACTGGGGAGTGGTTTTT
	LTR-II+III+IV M4+5	TTTTTGGGGACTTTCCAGGGAGGCGTGGCCTGTGCGTGACTGGGGAGTGGTTTTT
	LTR-II+III+IV random	AAAAACTACTGCACGCTCGCTACGACGACACTGTCGCGCATACAAGCTGCAAAAA
Taq polymerase stop assay	Taq Primer	GGCAAAAAGCAGCTGCTTATATGCAG
	LTR-II+III+IV	TTTTTGGGGACTTTCCAGGGAGGCGTGGCCTGGGCGGGACTGGGGAGTGGTTTTTCTGCATATAAGCAGCTGCTTTTTGCC
	LTR-II+III+IV M4+5	TTTTTGGGGACTTTCCAGGGAGGCGTGGCCTGTGCGTGACTGGGGAGTGGTTTTTCTGCATATAAGCAGCTGCTTTTTGCC
	LTR-III+IV	TTTTTGGGAGGCGTGGCCTGGGCGGGACTGGGGAGTGGTTTTTCTGCATATAAGCAGCTGCTTTTTGCC
	LTR-III	TTTTTGGGAGGCGTGGCCTGGGCGGGACTGGGGTTTTTCTGCATATAAGCAGCTGCTTTTTGCC
FRET	LTR-II+III+IV	d(6*FAM*-TGGGGACTTTCCAGGGAGGCGTGGCCTGGGCGGGACTGGGGAGTGGT-*TAMRA*)
	LTR-III+IV	d(6*FAM*-TGGGAGGCGTGGCCTGGGCGGGACTGGGGAGTGGT-*TAMRA*)

	LTR-III	d(6*FAM*-TGGGAGGCGTGGCCTGGGCGGGACTGGGGT-*TAMRA*)
	LTR-IV	d(6*FAM*-TGGGCGGGACTGGGGAGTGGT-*TAMRA*)
CD	LTR-II+III+IV	GGGGACTTTCCAGGGAGGCGTGGCCTGGGCGGGACTGGGGAGTGG
	LTR-III+IV	GGGAGGCGTGGCCTGGGCGGGACTGGGGAGTGG
	LTR-III	GGGAGGCGTGGCCTGGGCGGGACTGGGG
	LTR-IV	GGGCGGGACTGGGGAGTGG

Table S2. Energy Transfer (E), Energy Transfer Difference (ΔE) and Radius (Å) of LTRs in the absence or presence of hnRNPA2 or BSA, at different K^+ concentrations.

		**E**			**ΔE***			**R (Å)**		
**LTR**	**K^+ (mM)**	**G4**	**A2**	**BSA**	**G4**	**A2**	**BSA**	**G4**	**A2**	**BSA**
**II+III+IV**	*100*	0,84±0,002	0,53±0,02	0,82±0,002	0,727	0,309	0,018	38,1	49,1	38,9
	50	0,81±0,002	0,22±0,009	0,81±0,006	0,716	0,564	0,024	38,6	59,4	39,6
	25	0,83±0,003	0,26±0,02	0,80±0,0008	0,705	0,592	0,006	39,0	61,6	39,3
**III+IV**	*100*	0,72±0,006	0,50±0,01	0,70±0,002	0,709	0,222	0,017	42,8	50,2	43,4
	50	0,68±0,002	0,35±0,002	0,66±0,006	0,675	0,334	0,025	44,0	55,5	44,8
	25	0,65±0,002	0,34±0,02	0,63±0,005	0,646	0,317	0,028	43,6	56,0	45,9
**III**	*100*	0,80±0,004	0,73±0,006	0,77±0,012	0,781	0,066	0,021	39,6	42,2	40,8
	50	0,81±0,002	0,70±0,015	0,77±0,001	0,791	0,106	0,037	39,2	42,2	40,7
	25	0,81±0,006	0,66±0,01	0,77±0,002	0,786	0,141	0,033	39,4	44,6	40,8
**IV**	*100*	0,60±0,0007	0,46±0,01	0,53±0,02	0,452	0,141	0,053	46,6	51,3	48,9
	50	0,61±0,003	0,46±0,03	0,63±0,02	0,465	0,153	0,004	46,2	51,3	46,4
	25	0,61±0,002	0,39±0,01	0,57±0,003	0,461	0,218	0,039	46,4	53,8	47,6

*For free G-quadruplex, ΔE was calculated between the single-stranded oligonucleotide and the corresponding duplex.

Fig. S1. FRET-melting curves of untreated LTR-II+III+IV in the absence and presence of hnRNPA2 1:10, or of BSA as control, at a) K^+ 25 mM and b) 50 mM. c) and d) Corresponding first derivative curves, dF/dT versus T.

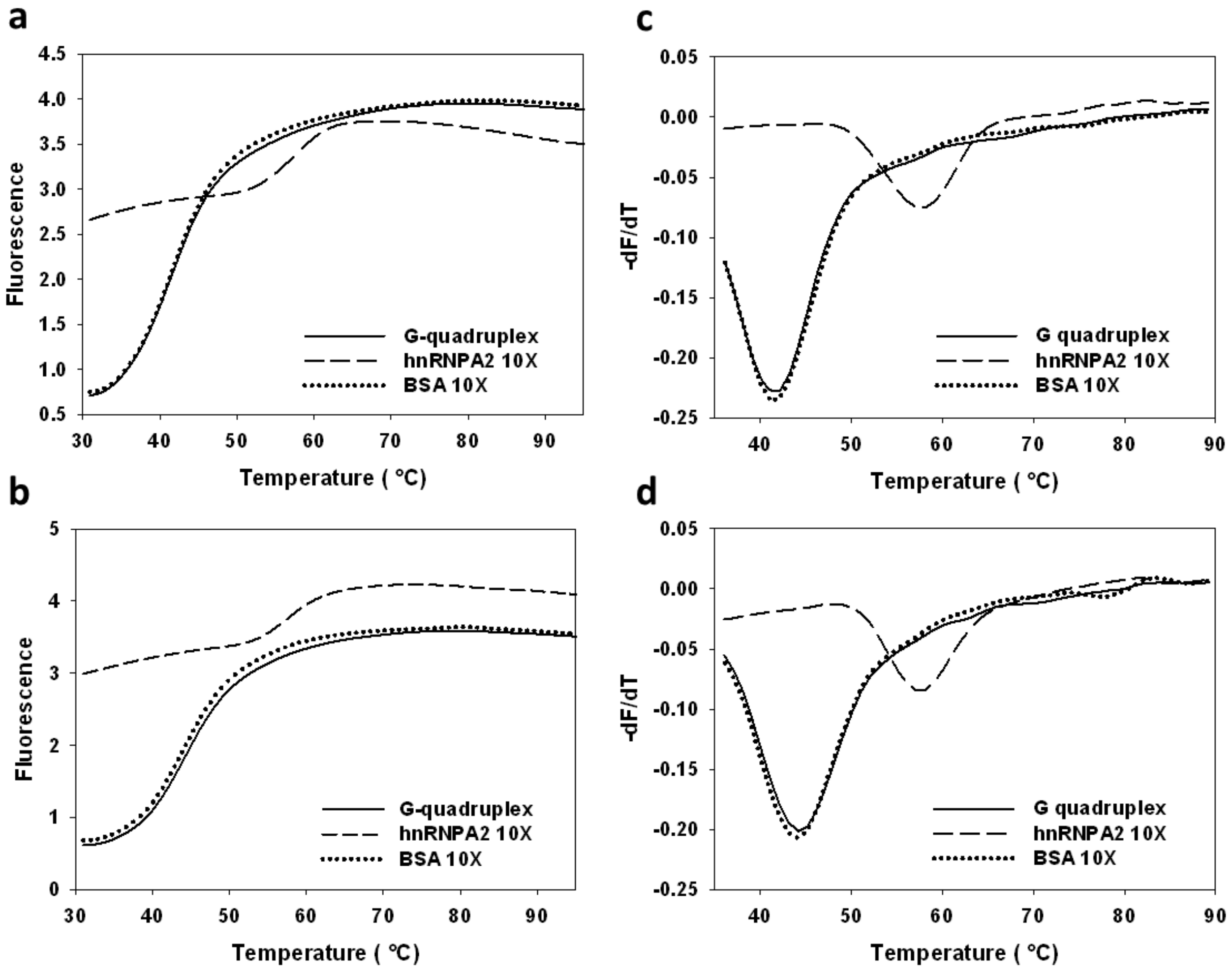

Fig. S2. FRET-melting curves of untreated LTR-III+IV in the absence and presence of hnRNPA2 1:10, or of BSA as control, at a) K^+ 25 mM, b) 50 mM and c) 100 mM. d), e) and f) Corresponding first derivative curves, dF/dT versus T.

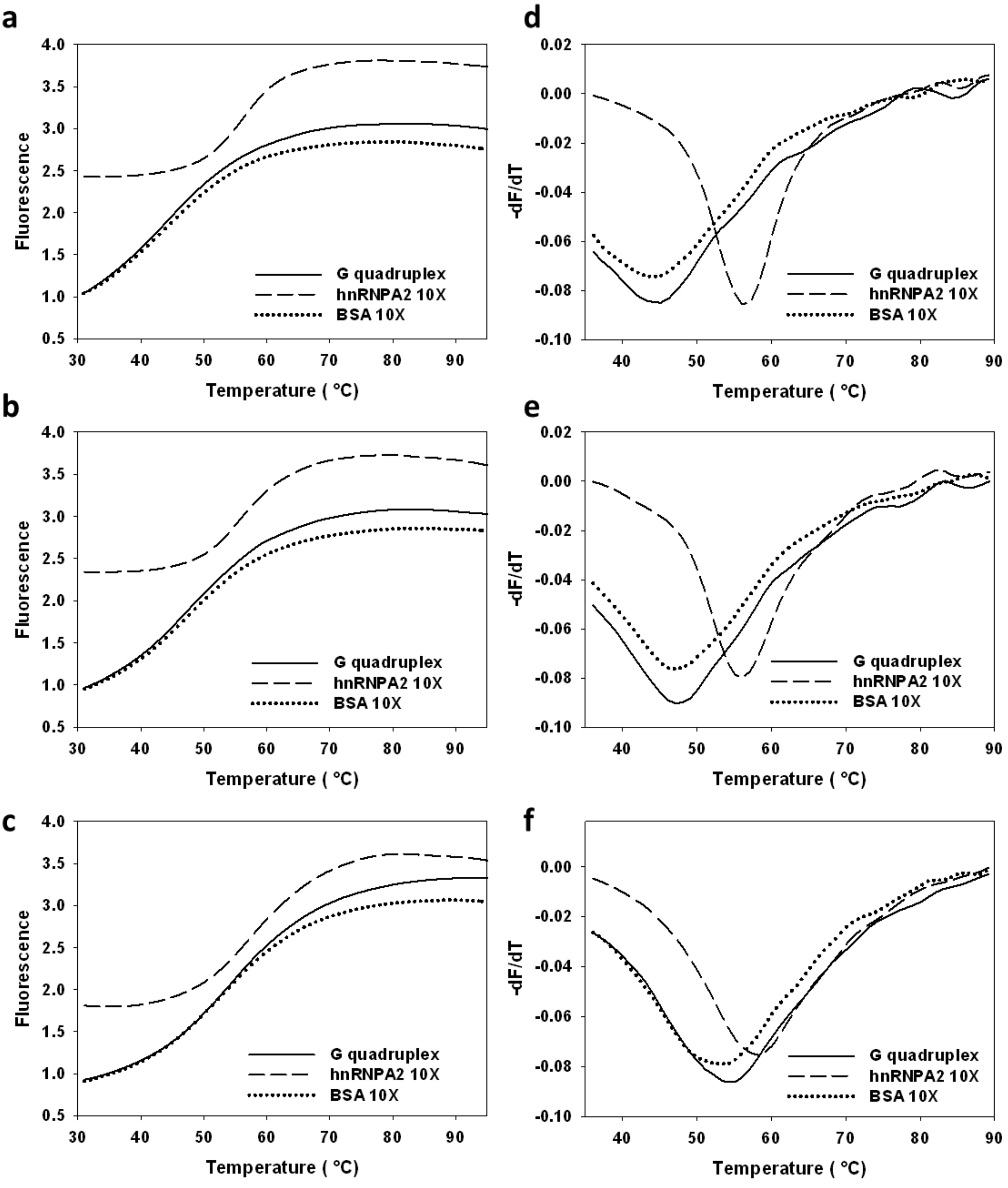

Fig. S3. A) FRET-melting curves of untreated LTR-III in the absence and presence of hnRNPA2 1:10, or of BSA as control, at a) K^+ 25 mM, b) 50 mM and c) 100 mM. d), e) and f) Corresponding first derivative curves, dF/dT versus T.

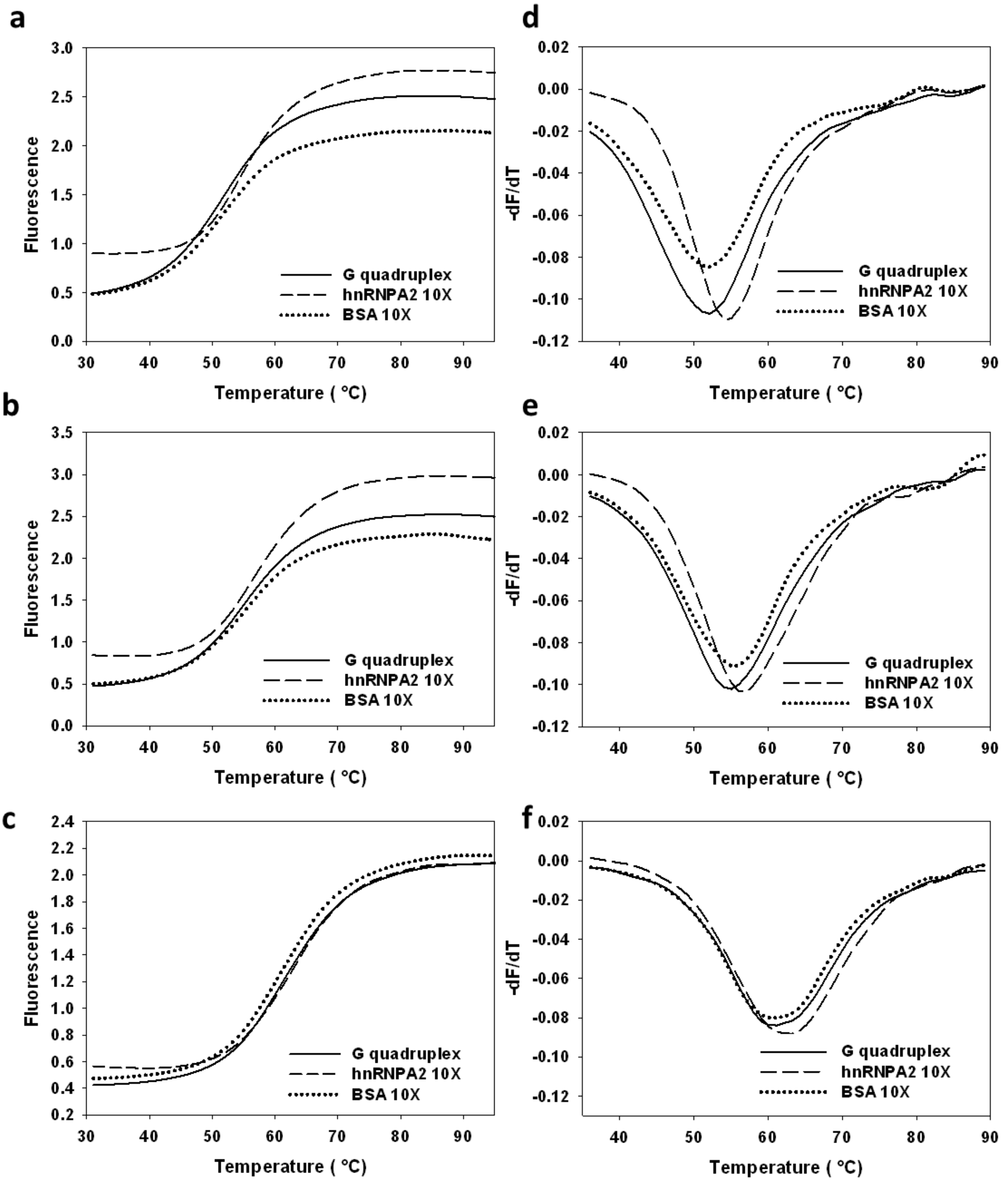

Fig. S4. A) FRET-melting curves of untreated LTR-IV in the absence and presence of hnRNPA2 1:10, or of BSA as control, at a) K^+ 25 mM, b) 50 mM and c) 100 mM. d), e) and f) Corresponding first derivative curves, dF/dT versus T.

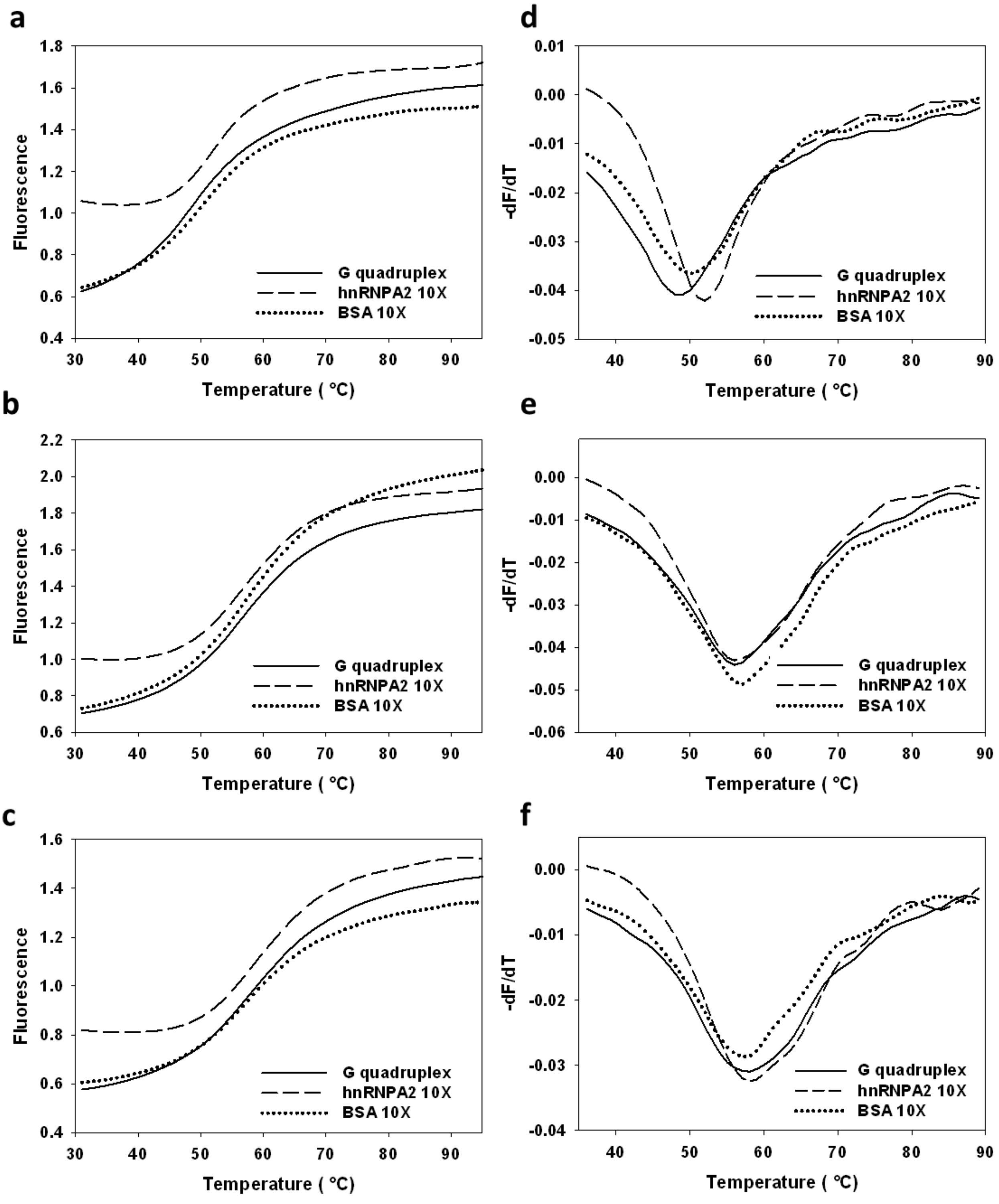

Fig. S5. Activity of hnRNP A2/B1 on the HIV-1 LTR promoter in TZM-bl cells. A) hnRNP A2/B1 depletion in TZM-bl cells by siRNAs analysed by western blot with anti hnRNP A2/B1 antibody. Scra indicates scrambled siRNAs. Detection of α-tubulin (αt) was used as control. NT indicates untreated cells. The symbol / indicates cells treated only with Lipofectamine RNAiMAX . B) Analysis of the luciferase activity of the LTR wt promoter in TZM-bl cells treated with hnRNP A2/B1 siRNAs, normalized to protein content.

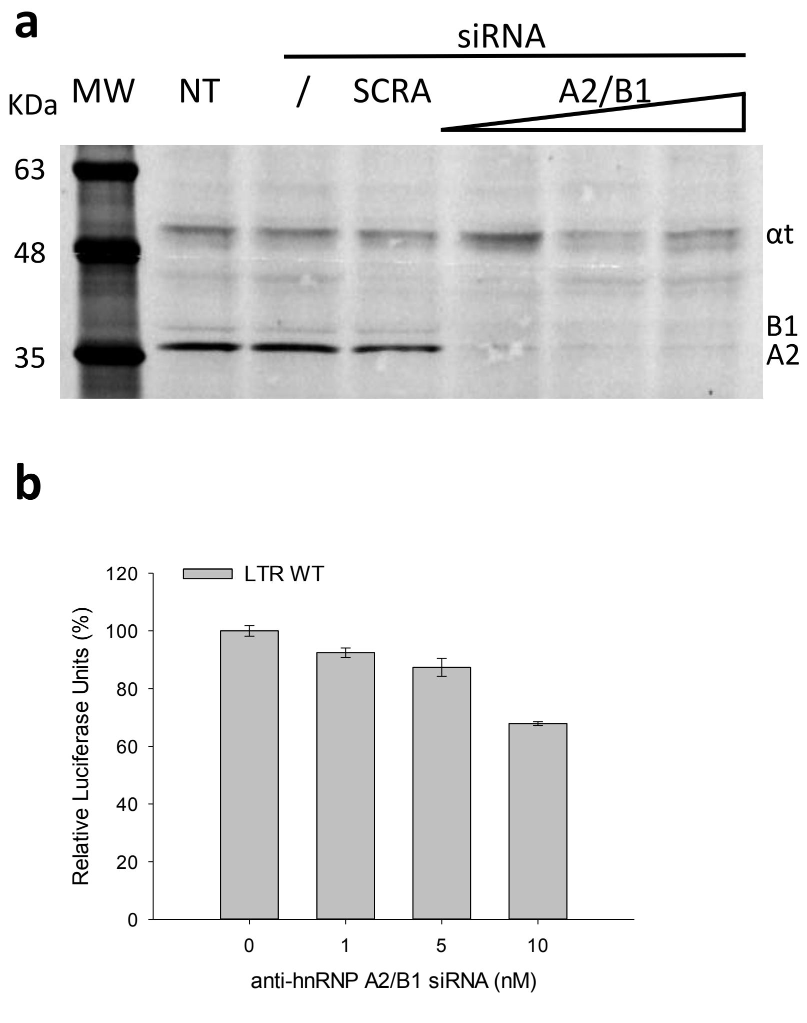

Supplement: Supplementary Information [file srep45244-s1.pdf]
